# A system for rating the stability and strength of medical evidence

**DOI:** 10.1186/1471-2288-6-52

**Published:** 2006-10-19

**Authors:** Jonathan R Treadwell, Stephen J Tregear, James T Reston, Charles M Turkelson

**Affiliations:** 1 ECRI Evidence-Based Practice Center and Health Technology Assessment Group, 5200 Butler Pike, Plymouth Meeting, Pennsylvania 19462, USA; 2 American Academy of Orthopaedic Surgeons, 6300 North River Road, Rosemont, Illinois 60018, USA

## Abstract

**Background:**

Methods for describing one's confidence in the available evidence are useful for end-users of evidence reviews. Analysts inevitably make judgments about the quality, quantity consistency, robustness, and magnitude of effects observed in the studies identified. The subjectivity of these judgments in several areas underscores the need for transparency in judgments.

**Discussion:**

This paper introduces a new system for rating medical evidence. The system requires explicit judgments and provides explicit rules for balancing these judgments. Unlike other systems for rating the strength of evidence, our system draws a distinction between two types of conclusions: quantitative and qualitative. A quantitative conclusion addresses the question, "How well does it work?", whereas a qualitative conclusion addresses the question, "Does it work?" In our system, quantitative conclusions are tied to *stability *ratings, and qualitative conclusions are tied to *strength *ratings. Our system emphasizes extensive *a priori *criteria for judgments to reduce the potential for bias. Further, the system makes explicit the impact of heterogeneity testing, meta-analysis, and sensitivity analyses on evidence ratings. This article provides details of our system, including graphical depictions of how the numerous judgments that an analyst makes can be combined. We also describe two worked examples of how the system can be applied to both interventional and diagnostic technologies.

**Summary:**

Although explicit judgments and formal combination rules are two important steps on the path to a comprehensive system for rating medical evidence, many additional steps must also be taken. Foremost among these are the distinction between quantitative and qualitative conclusions, an extensive set of *a priori *criteria for making judgments, and the direct impact of analytic results on evidence ratings. These attributes form the basis for a logically consistent system that can improve the usefulness of evidence reviews.

## Background

Systematic reviews, technology assessments, and clinical practice guidelines all incorporate evidence-based conclusions. The multifaceted nature of evidence, however, leads to varying degrees of confidence in how well the evidence supports conclusions drawn from it. For example, one is more confident in conclusions drawn from several well-designed randomized controlled trials that find similar effects than in conclusions drawn from a few poorly designed trials with disparate results. Consequently, methods for describing one's confidence in the available evidence are useful for end-users of evidence-based documents. This confidence is embodied in strength-of-evidence ratings.

In this article, we introduce a structured and transparent system for rating the strength of a body of evidence pertaining to a medical technology (see Note 1). We also define the concept of the *stability *of evidence as distinct from the *strength *of evidence. We identify the many judgments inherent in the process of performing evidence reviews, and then note how such judgments are incorporated within two prominent rating systems: the U.S. Preventive Services Task Force (USPSTF) system and the Grades of Recommendation Assessment, Development and Evaluation (GRADE) system [1-5]. Next, we describe our system, and detail several of its unique attributes. We present graphical illustrations of how our system provides a logical framework to combine the judgments inherent in evidence reviews. We then provide two complete examples to illustrate the system (one example for an intervention and one for a diagnostic test).

### Necessary Judgments

In performing an evidence review, an analyst must make numerous judgments about the available evidence. For many of these judgments, analysts at different centers could reasonably disagree about the status of the evidence. For example, one analyst may view a certain methodological flaw as fatal, whereas another analyst may view that same flaw as minor. This problem may be partially addressed through the use of standardized quality instruments. However, different centers tend to use different instruments, which can lead to different assessments of the quality of trials [6]. In the absence of empirical evidence on the extent to which a particular methodological flaw influences the results, the assessment of study quality necessarily entails judgment.

The challenge of conflicting judgment is magnified when several studies are available for review. With multiple studies, the analyst must also consider the degree of consistency among studies' results. Again, judgments must be made and different analysts may reasonably disagree. For example, different analysts may define "inconsistency" using different threshold values for I^2 ^(which is a statistical measure of the consistency of study results). Other components of the evidence analysis also require judgments, including quantity, robustness, and magnitude of effect. Definitions of these terms appear in Table 1, and additional judgments that are made during systematic reviews are listed in Table 2.

**Table 1:** A Guide to Terminology

**Major components of evidence**		
**Term**	**Definition**	

Quality	The extent to which studies are protected from bias	
Quantity	The number of studies and the number of patients	
Consistency	The extent to which different studies found similar results	
Robustness	The extent to which minor alterations in the data do not change conclusions drawn from that data	
Magnitude of effect	The effect size	

**Quantitative and qualitative conclusions**		

**Type of conclusion:**	**Quantitative**	**Qualitative**

Clinical interpretation:	How well does it work?	Does it work?
Type of rating:	Stability	Strength
Interpretation of rating:	Confidence that future evidence will not indicate a different effect size	Confidence that future evidence will not indicate a different direction of effect
Possible ratings:	High, Moderate, Low, or Unstable	Strong, Moderate, Weak, or Inconclusive

**Table 2:** Judgments Involved in Systematic Reviews

**Judgments pertaining to study quality assessment**
• What method will be used to assess study quality?
• If a quality scale is to be used, what scoring method will apply?
• What is the threshold for excluding a study from analysis due to poor quality?
• How will the individual study quality ratings be summarized to yield a single overall rating of quality to the evidence base (High, Moderate, or Low)?

**Judgment pertaining to sufficient evidence for quantitative estimate**

• What is the minimum number of studies required to permit a quantitative estimate?
• What is the minimum percentage of studies reporting accurate information (i.e., calculable effect sizes) required to permit a quantitative estimate?
• What imputation methods will be used for studies that did not report sufficient information for a calculable effect size?

**Judgments pertaining to initial meta-analysis**

• What effect size measure will be used?
• How will heterogeneity be measured (Q or I^2^)?
• What is the threshold for considering an evidence base heterogeneous?
• Will the summary effect size estimate be derived from a fixed-effects or random-effects meta-analysis?

**Judgments pertaining to quantitative robustness testing**

• What robustness tests will be used?
• If a cumulative meta-analysis is one of the robustness tests, will it be a fixed-effects or random-effects model?
• If a cumulative meta-analysis is one of the robustness tests, in what order will studies be entered into the cumulation?
• If a cumulative meta-analysis is one of the robustness tests, how many steps (i.e., study removals) will be examined to determine robustness?
• If a cumulative meta-analysis is one of the robustness tests, what threshold for a change in the summary effect size will be used to determine robustness?
• If publication bias testing is one of the robustness tests, which method of testing for publication bias will be used?
• If confidence interval width is one of the robustness tests, how narrow must the interval be for the summary estimate to be considered robust?
• Will overall robustness be judged based on passing all of the robustness tests, or simply a majority, or what percentage?

**Judgments pertaining to meta-regression**

• What is the minimum number of studies required to perform meta-regression?
• Which covariates will be included in multiple regression models?
• How many covariates are permitted in any given regression model?
• Does "explaining heterogeneity" require a statistically significant covariate, or the lack of resultant heterogeneity, or both of these?
• What robustness tests will be performed for the meta-regression?

**Judgments pertaining to qualitative robustness testing**

• What robustness tests will be performed?
• If a cumulative meta-analysis is one of the robustness tests, in what order will studies be entered into the cumulation?
• If a cumulative meta-analysis is one of the robustness tests, how many steps (i.e., study removals) will be examined to determine robustness?

**Other judgments**

• What is the size of a lowest possible effect that is still clinically important?
• What is the p value for statistical significance?
• What is the definition of a large magnitude of effect?
• How will qualitative consistency be defined (based on point estimates, confidence intervals, some percentage of studies, etc)?

In addition to these individual components (i.e., overall quality, quantity, consistency, robustness, and magnitude of effect of all evidence being considered), still another layer of judgment is required: how to combine each of these five components to produce a rating of the overall "strength" of the evidence. Different analysts may disagree about the relative importance of each component and their interplay. For example, if there is one small but well-conducted randomized controlled trial, how should one reconcile the high quality but low quantity of this evidence base to produce an overall strength rating? Or, how should one interpret very large effects observed in suboptimal study designs?

With all the judgments necessary, two different analysts, when faced with exactly the same clinical question and exactly the same evidence base, may have different degrees of confidence in their conclusions. In some instances, this discrepancy could be so large that the two analysts reach different overall conclusions. In a pilot study of inter-reviewer agreement, the GRADE group asked 17 experienced reviewers to rate the strength of the evidence for each of 46 outcomes within 12 systematic reviews [4]. Complete agreement occurred for only three outcomes (6%). The median kappa statistic of agreement was only 0.09, suggesting substantial inter-reviewer differences in evidence ratings. Although this study was conducted when the GRADE system was relatively new (and, therefore, these results may underestimate the true amount of agreement), the results do illustrate that even experienced reviewers can disagree about the strength of the evidence.

The subjectivity of judgments at several points in the systematic review process underscores the need for *transparency*. With transparent judgments, the end users of the review (including other analysts) can decide for themselves whether the judgments are reasonable.

### Currently Available Rating Systems

Numerous rating systems designed to assess the strength of a body of evidence have been proposed. Several of these were reviewed in a 2002 report from the Research Triangle Institute-University of North Carolina Evidence-based Practice Center (RTI-UNC EPC) [7]. This report focused on three components of a rating system: quality, quantity, and consistency. The report incorporated magnitude of effect within the quantity category, and did not mention robustness explicitly. Below, we highlight two major rating systems.

One prominent system is the Third U.S. Preventive Services Task Force (USPSTF) system [1]. This system, which is outlined in Table 3, employs ratings of the evidence at each of three strata: the individual study, the group of studies providing evidence on a single outcome, and the full body of evidence on all outcomes. If the overall rating at the third stratum is Good or Fair (i.e., at least some net benefit), the USPSTF system then incorporates magnitude of effect, separately for benefits and harms. Here, the goal is to weigh the relative benefits and harms in order to estimate the overall net benefit of the technology. Such weighting requires yet another layer of judgment: the relative importance of several different outcomes. One criticism of the USPSTF system, and several similar systems, is the lack of transparency in judgments [2]. Although the USPSTF system lists numerous factors one should consider when making judgments, and narrative text is used to explain these judgments, there is no formal mechanism (e.g., a point system) that would allow end users to reproduce these judgments. Furthermore, the manner in which judgments are combined in the USPSTF system is not specified.

**Table 3:** USPSTF System for Evaluating the Quality of Evidence (Harris et al. 2002)[1]

**Level of evidence: Individual study**
**Criteria for judging quality**:
• Internal validity – based on a series of separate, specific criteria for systematic reviews, RCTs, cohort studies, case-control studies, and diagnostic accuracy studies
• External validity – degree to which the study is generalizable to the population of interest and conditions of typical clinical practice

**Level of evidence: Linkage (key question) in the analytic framework**

**Criteria for judging quality:**
• Aggregate internal validity of studies addressing the linkage
• Aggregate external validity of studies addressing the linkage
• Coherence/consistency of studies addressing the linkage

**Level of evidence: Entire preventive service**

**Criteria for judging quality**:
• Quality of the evidence for each linkage in the analytic framework
• Degree to which a complete chain of linkages supported by adequate evidence connects the preventive service to health outcomes
• Degree to which the complete chain of linkages "fit" together ^a^
• Degree to which the evidence connecting the preventive service and health outcomes is "direct" ^b^

^a^"Fit" refers to the degree to which the linkages refer to the same population and conditions. For example, if studies of a screening linkage identify people who are different from those involved in studies of the treatment linkage, the linkages are not supported by evidence that "fits" together.

^b^"Directness" of evidence is inversely proportional to the number of bodies of evidence required to make the connection between the preventive service and health outcomes. Evidence is direct when a single body of evidence makes the connection, and more indirect if two or more bodies of evidence are required.

One major effort to create a formal point system is the GRADE system [2-5] The GRADE approach (Table 4) emphasizes the primacy of study design, which is used to set a starting quality grade. Then, other components are considered which may increase or decrease the grade (see Table 4 under quality of evidence for each outcome). The GRADE approach results in one of four outcome-specific grades: High, Moderate, Low, and Very Low. Definitions of these terms are based on the likelihood that further evidence will change one's confidence in the size of the effect. The American College of Chest Physicians Task Force recently described the use of a revised GRADE system that combined Low and Very Low into one category (Low) [5].

**Table 4:** The GRADE System for Grading Quality of Evidence and Strength of Recommendations[2,5]

**Quality of evidence for each outcome (high, moderate, or low) **– based on the following criteria:
**• Study design**– grade of high or low assigned based on design (RCT = high, observational study = low)
**• Study quality**– Detailed study methods and execution.
1) Limitations in quality can decrease grade one or two levels.
2) Evidence of reporting bias can also decrease grade one level.
3) Grade can be increased one level if all plausible confounders would have reduced the treatment effect.
**• Consistency of results**– The level of similarity of estimates of effects across studies.
1) Important inconsistency can decrease grade one level.
**• Directness of evidence**– the extent to which the people, interventions, and outcome measures are similar to those of interest.
1) Some or major uncertainty about directness lowers the grade one or two levels.
**• Other considerations**
1) Magnitude of effect can increase the grade of evidence. Strong evidence of association (significant relative risk of >2 or <0.5 based on consistent evidence from 2 or more observational studies with no plausible confounders) increases the grade by one level. Very strong evidence of association (significant relative risk of >5 or <0.2 based on direct evidence with no major threats to validity) increases the grade by two levels.
2) Evidence of a dose-response gradient increases the grade by one level.
3) Imprecise or sparse data can lower the grade by one level.
**Relative importance of outcomes **– included outcomes should be critical or important (but not critical) to a decision
**Overall quality of evidence **– judged across outcomes based on the lowest quality of evidence for any of the critical outcomes
**Balance of benefits and harms **– classified as net benefits, trade-offs, uncertain trade-offs, or no net benefits based on the important health benefits and harms
**Balance of net benefits and costs **– are incremental health benefits worth the costs?
**Strength of recommendation **– the extent to which one can be confident that adherence to a recommendation will do more good than harm

Within the various increments and decrements in GRADE, several implicit judgments are necessary. For example, what is a "serious" limitation in study quality or an "important" inconsistency among effect sizes? Such judgment is an inevitable part of performing systematic reviews, as discussed earlier, and the GRADE group readily acknowledges the need for judgment. Further, a key motivation behind the GRADE effort was a need for "explicit definitions" and "sequential, explicit judgments,"[2] and the GRADE system represents an importance advance towards transparency.

An explicit system for combining these judgments (i.e., a point system) is a particular strength of the GRADE approach. This system defines precisely how the various aspects of the evidence are combined to arrive at an overall grade of the evidence for each outcome. Thus, if a user made different judgments than the analyst, then the user could apply the GRADE point system accordingly, and possibly arrive at a different rating of the evidence. For example, suppose an analyst judged that there were important inconsistencies in the data for a certain outcome, but a user examined the same data and believed that the inconsistency was unimportant. Using the GRADE system, the user could then increase the final grade by one level.

In addition to USPSTF and GRADE, several other prominent systems for rating the strength of evidence have been proposed by the American College of Chest Physicians (ACCP) [8], the Australian National Health and Medical Research Council (ANHMRC) [9], the Oxford Centre for Evidence-Based Medicine (OCEBM) [10], the Scottish Intercollegiate Guidelines Network (SIGN) [11], and the U.S. Task Force on Community Preventive Services (USTFCPS) [12]. Each of these systems were reviewed by the GRADE group, which concluded that these systems all have important shortcomings [2].

## A New System

**Figure 1:**
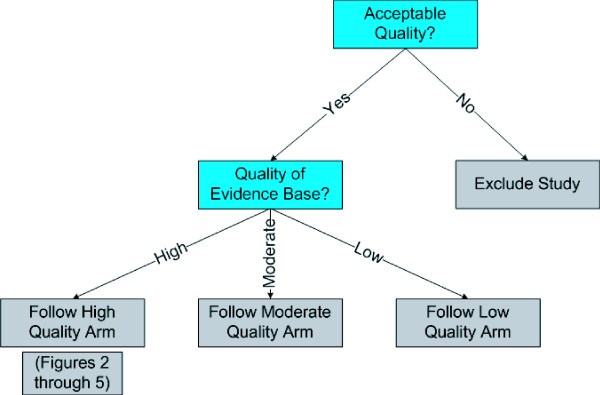
Entry Into System.

**Figure 2:**
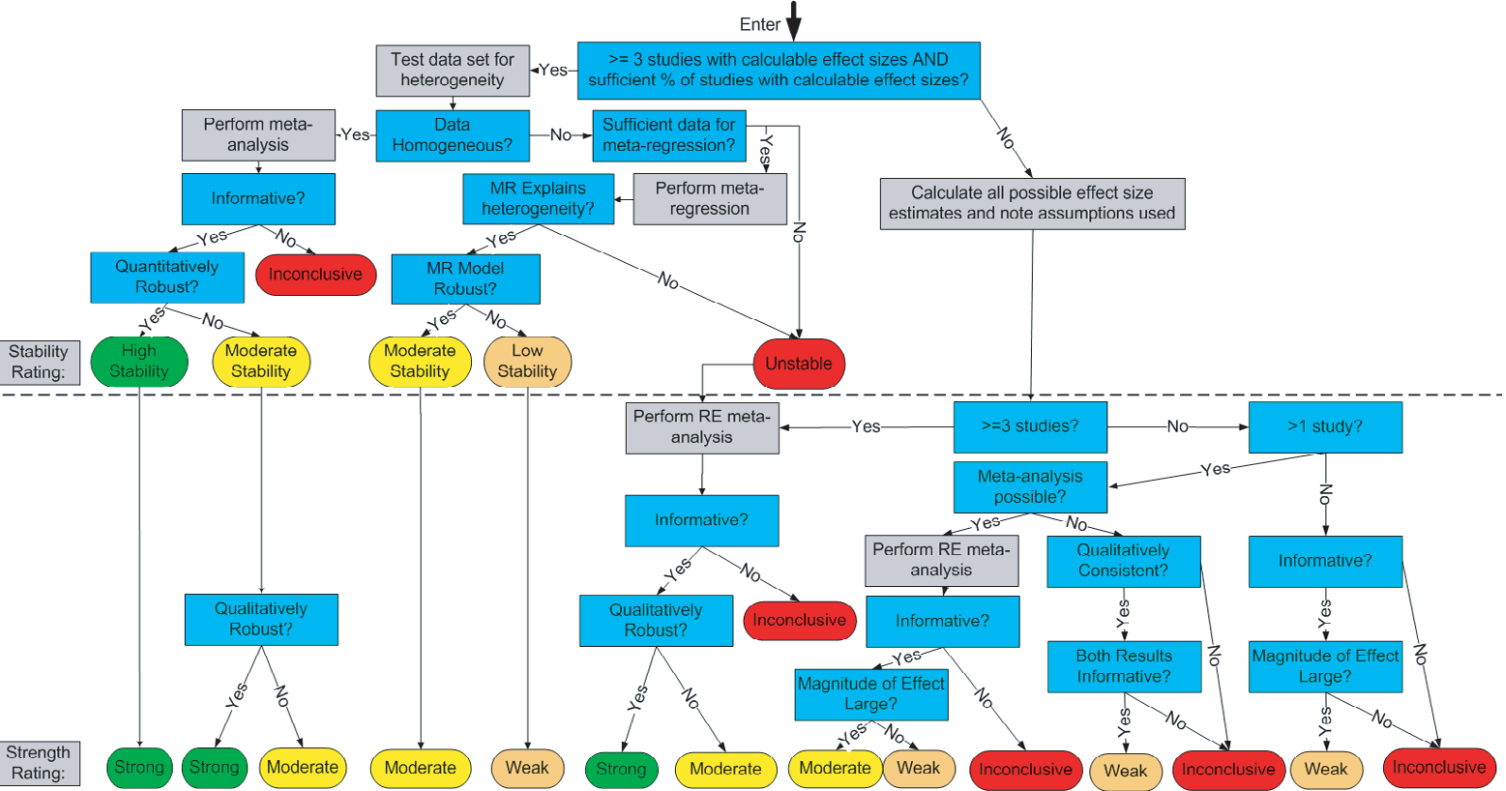
Overview of the High Quality Arm.

**Figure 3:**
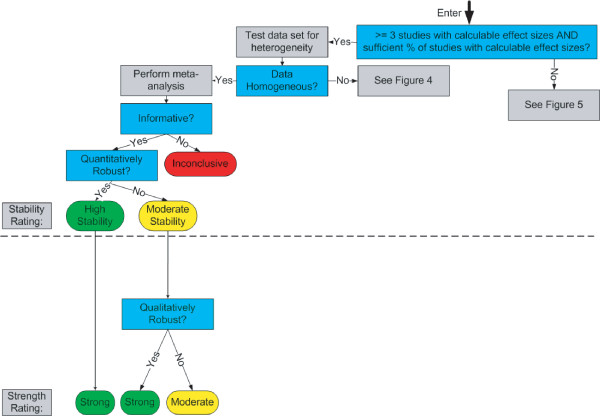
High Quality Arm: Homogeneous Data.

**Figure 4:**
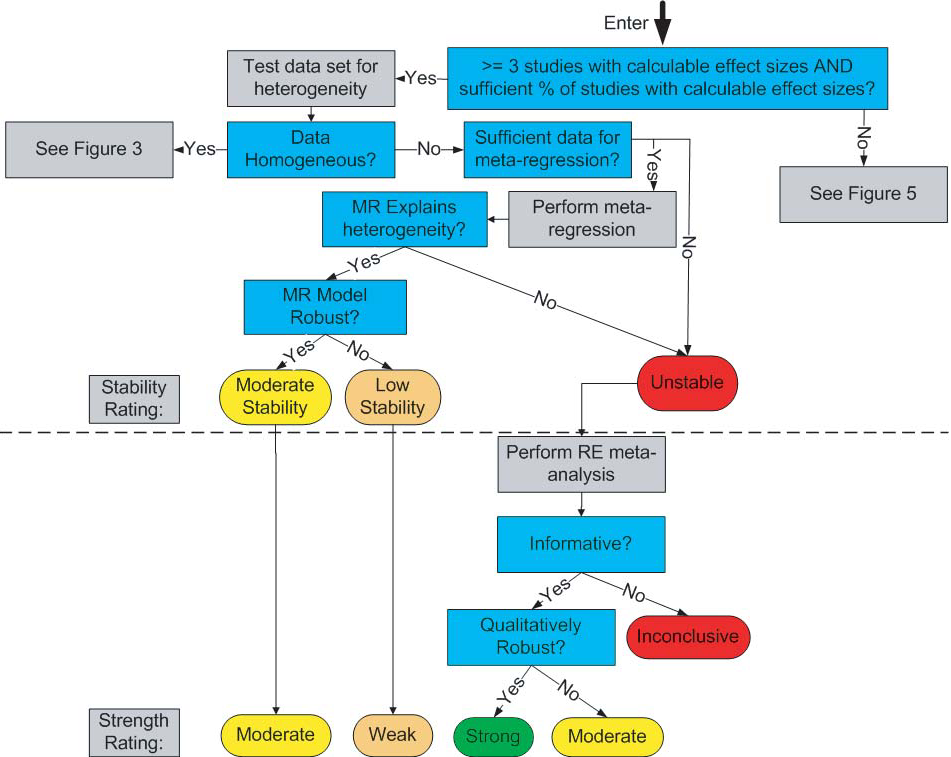
High Quality Arm: Heterogeneous Data.

**Figure 5:**
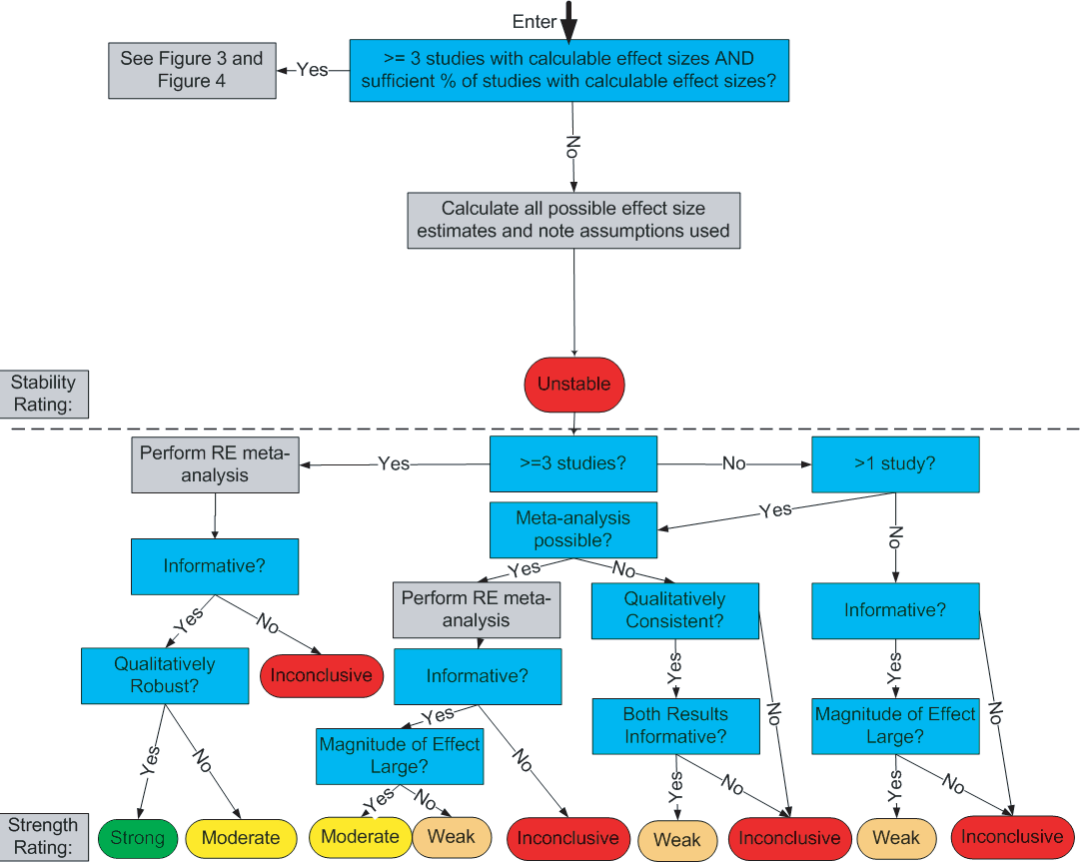
High Quality Arm: Small Evidence Base.

We now describe a new system for rating the strength of evidence. Like the GRADE system, our system emphasizes the need for making judgments explicit, and uses the equivalent of a formal point system for rating the strength of a body of evidence. Our system, however, is unique in several fundamental respects. Below, we describe the three prominent areas of uniqueness: 1) the distinction between quantitative and qualitative conclusions; 2) extensive use of *a priori *criteria for judgments; and 3) the direct impact of meta-analysis and sensitivity analysis on evidence ratings. We then provide details on the system itself, including graphical depictions of how judgments can be combined (Figure 1, Figure 2, Figure 3, Figure 4, and Figure 5).

Our system is designed to rate the stability and strength of evidence for each outcome an analyst chooses to evaluate (see Note 2), and is not intended to produce overall recommendations for (or against) a technology. By contrast, both USPSTF and GRADE rate the strength of the *overall evidence for the technology *(which is often based on several outcomes), and the strength of a *recommendation about the technology*. The first involves assessing the overall net benefit by balancing the benefits and harms. The second entails more judgments (listed in the USPSTF methods article) about the importance and impact of cost, ethics, law, patient expectations, and societal expectations [1]. Also, unlike GRADE and USPSTF, our evidence rating system focuses on only the *internal validity *of the evidence. Questions about generalizability can be addressed outside the scope of the rating system. We are currently examining how well our evidence rating system can be applied within the overall GRADE framework.

### Quantitative and Qualitative Conclusions

Our system draws a distinction between two types of conclusions: quantitative and qualitative (see the bottom half of Table 1). The quantitative conclusion addresses the question, "How well does it work?", and we refer to the corresponding rating as a "stability" rating. By contrast, the qualitative conclusion addresses the more general question, "Does it work?", and we refer to the rating of the evidence pertaining to this conclusion as a "strength" rating. Thus, a quantitative conclusion characterizes the size of the effect, whereas a qualitative conclusion characterizes the direction of the effect.

This key distinction allows one to draw a strong qualitative conclusion in the face of quantitatively heterogeneous data. Such a situation arises when the results of all studies included in an evidence base demonstrate efficacy, but the magnitude of measured treatment effect differs considerably across studies.

**Figure 6:**
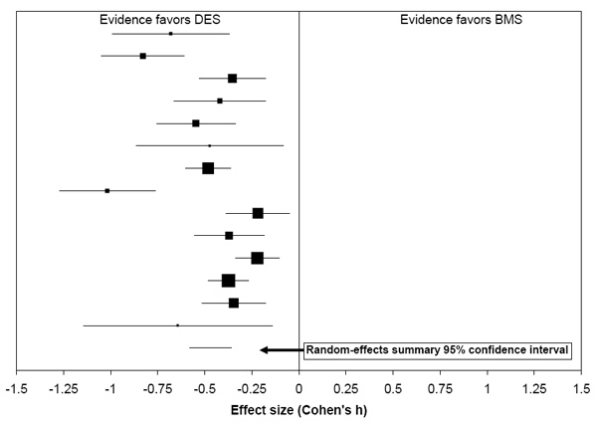
**Forest Plot Demonstrating the Quantitative/Qualitative Distinction**. Plot showing the results of 14 randomized trials that compared drug-eluting stents (DES) to bare metal stents (BMS) and reported the percentages of patients who underwent target lesion revascularization after stent implantation. Sizes of the squares are proportional to study size, and 95% confidence intervals are shown as horizontal lines. There was unexplained heterogeneity among the trial results, so we did not estimate the size of the difference between groups. However, the random-effects meta-analytic confidence interval at the bottom of the plot showed the summary statistic was statistically significant.

This situation is illustrated in a recent systematic review on drug-eluting stents for coronary artery disease [13]. This review included 14 randomized trials that compared drug-eluting stents to bare metal stents. Each trial reported the percentage of patients in each arm who underwent target lesion revascularization (TLR) after stent implantation (Figure 6). A homogeneity test of these data identified substantial heterogeneity among trial results (Q = 59, p < 0.0001, I^2 ^= 78%), and subsequent meta-regression analyses did not explain this heterogeneity. Consequently, we refrained from presenting an estimate of the size of treatment effect. However, all trials found that TLR rates were lower after implantation of a drug-eluting stent than after a bare metal stent, and the random-effects meta-analytic confidence interval demonstrated this clear direction of effect (see the bottom of Figure 6). Thus, although one can have little confidence in the accuracy of a single quantitative estimate of the effect size, one can have high confidence that drug-eluting stents are effective in reducing TLR rates. Thus, the quantitative/qualitative distinction also underpins two notions of *consistency*. The first is quantitative consistency: do the studies report similar effect sizes? The second is qualitative consistency: do the studies report the same direction of effect?

Another important purpose of differentiating quantitative from qualitative conclusions is to acknowledge the different needs of those who utilize systematic reviews. Some users are primarily interested in obtaining an estimate of the amount of benefit (or harm) associated with a technology. Other users are simply interested in whether the technology provides any benefit at all. If a systematic review provides both kinds of conclusions, then both needs are met.

No other system for rating medical evidence distinguishes explicitly between quantitative and qualitative conclusions. We believe the distinction is critical to ensure that systematic reviews provide a full picture of the evidence. The GRADE group defines their ratings as the likelihood that further evidence will change one's confidence in the size of the effect, which appears to be a purely quantitative definition. The USPSTF system does not state whether its ratings refer to quantitative conclusions, qualitative conclusions, or both.

In our system, stability and strength ratings are not independent. Logically, evidence that permits a "highly stable" estimate of treatment effect (e.g., an odds ratio of 3.25 in favor of treatment) must also permit a "strong" conclusion about the direction of effect (e.g., that the odds ratio favors treatment). Thus, one built-in feature of our system is that the stability rating sets a lower bound on the strength rating. This means that "moderate" stability can be accompanied by a strength rating no lower than "moderate", and "low" stability can be accompanied by a strength rating no lower than "weak."

Crucial to understanding the results of a systematic review is understanding whether the results are clinically important; statistically significant results do not necessarily represent a clinically important effect. This has been mentioned in many systematic reviews [14-18], and our quantitative/qualitative distinction provides an analytic approach. To address clinical importance in our system, one first defines precisely the magnitude of effect that is considered clinically important (e.g., a difference of 0.5% in H_b_A_1c _in treatments for diabetes). Then, clinical importance can be addressed as a qualitative question: "Is the difference clinically important?" This question is addressed analytically via a comparison of effect sizes to an effect size predefined as clinically important.

### Extensive *A Priori *Criteria for Judgments

Most systematic reviews use *a priori *inclusion criteria to reduce the potential for bias in judgments about which studies to include. Some also make an *a priori *judgment about which instrument will be used to assess study quality. However, many other judgments are still susceptible to bias. To reduce this potential, our system specifies the use of *a priori *judgments wherever possible. For example, the system requires that one specify *a priori *quantitative definitions of "consistent" and "robust" effects. Also, the analyst must pre-specify the minimum percentage of included studies that reported the outcome of interest in order to permit a meta-analytic estimate of effect size. If only a small percentage of included studies reported the outcome, selective outcome reporting may have occurred, thereby biasing the meta-analytic summary statistic. For study quality, the analyst must identify not only the instrument to be used, but also the scoring system (if used) and the thresholds that define study quality categories (high, moderate, or low quality). Even the threshold for statistical significance (which does not have to be the conventional 0.05 in all contexts because some clinical contexts may warrant greater or lesser concern about Type 1 errors) must be specified beforehand. To address the question of clinical importance, the minimum level considered to be clinically important must also be determined *a priori*. These definitions, and others, are discussed further in the section entitled "How the System Works".

### Consequences of Meta-Analysis and Sensitivity Analysis

Many systematic reviews report the results of meta-analyses, and some also describe sensitivity analyses. Often, however, the results of these statistical analyses are not explicitly tied to ratings of the evidence. In this section, we describe how our system links analytic results to both stability and strength ratings.

The purpose of meta-analysis is not just to obtain a summary estimate of treatment effect, but also to test the data for consistency (heterogeneity testing). This latter purpose is typically accomplished using the Q-statistic, and more recently, I^2 ^[19,20] If important heterogeneity is detected, our system requires that, whenever appropriate (e.g., when the evidence base is large enough), the analyst explore potential sources of this heterogeneity using meta-regression. If heterogeneity cannot be explained by meta-regression, then our system precludes one from presenting a single summary estimate of treatment effect (i.e., a stability rating of "Unstable") (see Note 3). Some investigators advocate the use of a random effects summary statistic in this situation. However, unexplained heterogeneity could be due to differences in patient populations, and/or the way a treatment is administered. Our view is that computing a *single *summary estimate is not warranted when the evidence demonstrates the existence of *multiple *estimates.

Although our system precludes the use of random-effects models in determining a single summary estimate of treatment effect, the use of these models does have an important role. This role involves a summary of the evidence to support a *qualitative *conclusion. Even if there is substantial unexplained heterogeneity, the evidence may still indicate a consistent *direction *of effect. The confidence interval (CI) around the random effects summary statistic, which incorporates both within-study and between-study variance, may lie fully above 0 or below 0 (see Note 4). This CI, therefore, is suitable for determining whether the data suggest a clear direction of effect.

Other systems, such as GRADE and USPSTF, are largely silent on the role of meta-analysis in systematic reviews. Our system uses meta-analysis and meta-regression (when clinically appropriate) to increase statistical power and employ precise study weights; furthermore, the system is unique in incorporating the results of these analyses into evidence ratings.

We now turn to the role of sensitivity analysis in our system. In this context, consider that the goal of rating evidence is to assess the likelihood that future evidence will indicate something different than what current evidence indicates. If a large amount of consistent evidence has already accumulated, then future evidence is unlikely to alter the overall strength or stability. Conversely, conclusions based on only a small amount of accumulated evidence may easily change when a single new study is published.

Considered from this perspective, we argue that sensitivity analysis (see Note 5) can substitute for certain judgments about quantity. The idea is that if the conclusion from a meta-analysis depends critically on only one or a few studies in that analysis (or if there is reason to suspect that not all relevant studies are available), then the conclusion may not be robust. Such dependence suggests that a future study may alter conclusions based on currently available studies. Consequently, our system downgrades the stability or strength ratings accordingly. Although there is a widespread sense that sensitivity analysis should be incorporated into an analysis, the system is unique in offering explicit rules for how to gauge the impact of the results of sensitivity analyses on one's confidence in the available evidence.

Sensitivity analysis can obviate the need for certain subjective judgments about the magnitude of effect. Some rating systems (e.g., GRADE) employ such judgments, and if the observed effect is very large, the evidence receives a higher strength rating. Presumably, this is because a very large effect is less likely to be overturned by future evidence and is therefore more robust. However, if there are sufficient studies to perform direct robustness tests via sensitivity analyses, then we advocate doing so, in lieu of making judgments about effect sizes. A meta-analytic sensitivity analysis incorporates effect sizes and confidence intervals from all studies, so that the test is empirically-based.

As with consistency, the quantitative/qualitative distinction helps clarify two notions of robustness. Quantitative robustness concerns the degree to which the summary effect size from meta-analysis tends to change based on relatively small alterations in the data. To assess quantitative robustness, one can perform successive meta-analyses and observe the relative changes in the summary estimate. If the changes in the estimate exceed a predetermined tolerance level, then the original summary estimate is not quantitatively robust. Qualitative robustness refers to whether the evidence base yields the same qualitative general conclusion upon alterations of the data. To assess it, one can again perform successive meta-analysis, but in this case the issue is whether the confidence intervals around summary statistics consistently indicate the same direction of effect.

**Figure 7:**
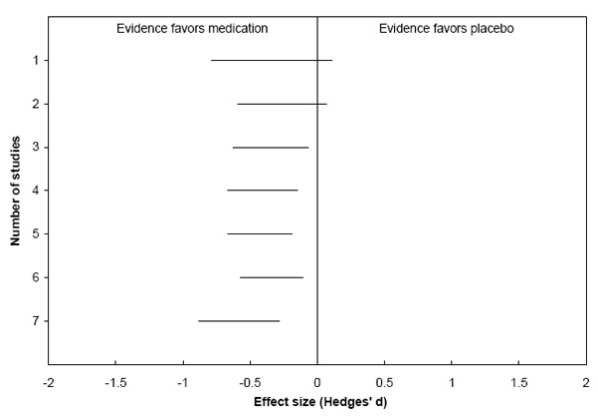
**Example of a Qualitative Robustness Test**. Cumulative meta-analytic-test of the qualitative of seven randomized trials that compared pharmacotherapeutic treatment for bulimia to placebo and reported mean purging frequency. We performed a cumulative meta-analysis in which the study with the largest weight was entered first (the topmost horizontal segment in the plot), and then the study with the next largest weight was entered (the second one from the top), etc. Each horizontal segment in the plot is a 95% confidence interval around a random-effects summary Hedges' d, a standardized mean difference. (The point estimates are not shown to clarify that the analysis focuses only on confidence intervals, not point estimates). In each of the last five analyses, the effect was statistically significant in the same direction. This met our a priori definition of qualitative robustness, which was that the qualitative conclusion must have remained the same after each of the last three or more studies were added.

For example, one qualitative robustness test we have employed utilized cumulative meta-analysis [21]. In a report on treatments for bulimia [22], we included seven randomized trials that compared the efficacy of pharmacotherapy to placebo and reported mean purging frequency. A random-effects meta-analysis found that medication yielded significantly greater effects than placebo (i.e., lower purging frequency). We tested the qualitative robustness of this finding in the following manner (see Figure 7). The 95% confidence interval of the study with the largest weight (as determined by the inverse of the variance) in the meta-analysis was plotted first (the topmost horizontal segment in the figure). Then we added the study with the next largest weight, and plotted the corresponding random-effects 95% confidence interval for the two-study meta-analysis (the second segment from the top in the figure). Then we continued adding studies, one at a time, until all meta-analytic confidence intervals were plotted. *A priori*, we had defined a qualitative robust evidence base as one where each of the last three cumulative meta-analyses yielded the same qualitative conclusion. Therefore, we deemed this evidence base to be qualitatively robust.

### How the System Works

The system is shown graphically in five figures:

• Entry into system (Figure 1)

• Overview of the high quality arm (Figure 2)

• Homogeneous data (Figure 3)

• Heterogeneous data (Figure 4)

• Small evidence base (Figure 5)

An important feature of this system is that every question illustrated in the figures requires a set of *a priori *criteria. Nearly all of these *a priori *criteria are operational definitions that are quantitative. The use of *a priori *criteria helps to reduce bias and subjectivity, as discussed above, and the use of quantitative definitions increases transparency. This system assumes that the assessor has already applied appropriate inclusion/exclusion criteria and has excluded from the analysis any study with fatal flaws.

The initial entry into the system occurs with an assessment of the quality of the evidence for a specific outcome (Figure 1), which we consider to be the most important aspect of the evidence. Quality sets an upper bound on the stability and strength ratings (e.g., moderate strength is only possible for data that is, at minimum, moderate quality). Although quality evaluation can be performed with a checklist or scale, any reasonable method for separating the evidence base into different categories of quality will suffice. After an evaluation of individual study quality, studies are judged to be high, moderate, low, or very low quality. Studies of very low quality are always excluded from the evidence base, and the analyst may also choose to exclude low or even moderate quality studies as well. The analyst must choose a method for aggregating the quality of the individual studies to obtain an overall quality rating for the evidence base and then enter the high, moderate, or low quality arm of the system. Within these arms, the system further assesses the quantity, consistency, robustness, and (in some instances) magnitude of effect to determine the stability and strength of the evidence.

Figure 2 through Figure 5 detail the high quality arm of the system. The top half of each figure includes all of the questions and decisions that impact stability ratings (and quantitative conclusions), while the bottom half includes all of the questions and decisions that impact strength ratings (and qualitative conclusions). The moderate and low quality arms of the system are not shown because all aspects of this system are already displayed in the high quality arm.

At the top of these pathways, one first considers whether the evidence base is sufficient to provide a single quantitative estimate of the effect size. We generally require at least three studies, but other investigators may wish to set this criterion higher (e.g., five studies). Additionally, the system requires that a certain percentage of the studies (e.g., 80% or more) must have calculable effect sizes (that can be determined without imputation). If these criteria *are not met*, then one proceeds to Figure 5 (small evidence base). If these criteria *are met*, then one tests the quantitative consistency of the data using a heterogeneity measure such as Q or I^2^. Under homogeneity, one proceeds to Figure 3, whereas under heterogeneity, one proceeds to Figure 4.

**Figure 8:**
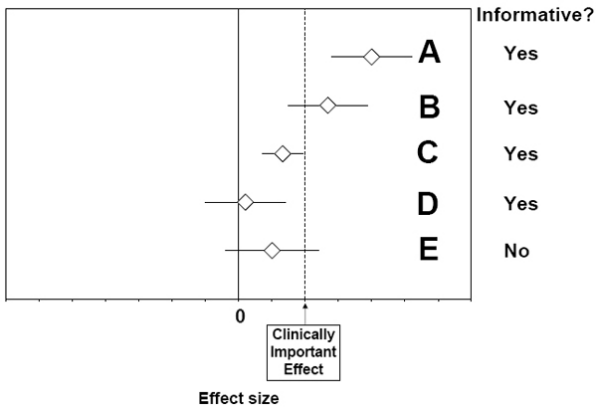
**Informative and Non-Informative Effect Sizes**. This figure is adapted from Armitage and Berry.[23] Each open diamond denotes a hypothetical meta-analytic summary statistic, and the horizontal segments denote 95% confidence intervals. The dashed vertical line indicates the effect size that was determined a priori to represent the minimum effect size that is considered clinically important. A meta-analytic summary statistic is considered informative if its confidence interval either excludes 0 or excludes a clinically important effect (or both). Thus, meta-analyses A through D each show informative results, whereas meta-analysis E shows a non-informative result.

Before detailing the steps in Figures 3 and 4, we must first define the concept of "informativeness", a concept crucial to interpreting the results of individual studies and meta-analyses. Figure 8 illustrates four different effect sizes (A through D) that are considered informative based on criteria discussed in Armitage and Berry [23]. These effects are informative because the confidence intervals around the summary effect estimates support one of four qualitative conclusions: A) the treatment is beneficial *and *the effect is clinically important (i.e., the lower 95% confidence interval around the meta-analytic summary statistic is greater than the effect size deemed clinically important); B) the treatment is beneficial but the effect may or may not be clinically important (i.e., the lower 95% confidence interval around the meta-analytic summary statistic is greater than zero but less than a clinically important effect); C) the treatment is beneficial but the effect is not clinically important (the 95% confidence interval is between zero and the effect deemed clinically important); or D) the treatment is not beneficial (the 95% confidence interval overlaps zero and does not overlap the line of clinical importance) (see Note 6). By contrast, example E in Figure 8 would be considered inconclusive (non-informative) because the 95% confidence interval overlaps both zero and the line of clinical importance. Note that this use of "informativeness" accounts for the statistical power of the evidence base, another unique feature of our system (for a related discussion see Armitage and Berry) [23]. Moreover, by incorporating clinical importance into the system, we provide clinical meaning for the end-users of systematic reviews and other evidence-based documents.

In the homogeneous pathway (Figure 3), one performs a meta-analysis to combine the study results. If the meta-analytic summary statistic is not informative, then no conclusions are reached. If the summary statistic is informative, one tests the robustness of the findings through sensitivity analysis (e.g., removal of one study at a time). If the meta-analytic summary statistic passes the robustness tests, the estimate is quantitatively robust. This produces a high stability rating for the quantitative estimate, which leads directly to a strong qualitative conclusion. The logic behind this implication is that if one is confident in the specific estimate of the effect, one is automatically confident in the general direction of that effect.

Continuing within Figure 3, if the findings are not quantitatively robust, one re-examines the sensitivity analyses to determine qualitative robustness (e.g., do any of the last three analyses in a cumulative meta-analysis of a given data set lead to a different qualitative conclusions?). Additional sensitivity analyses that can be used include removing each study separately or changing the effect size statistic (e.g., using Cohen's h instead of an odds ratio). We also consider tests for publication bias to be a form of sensitivity analysis, although publication bias testing requires a minimum number of available studies. Whether the findings are qualitatively robust determines whether one reaches a strong or moderate qualitative conclusion. Also, one can only reach a strong conclusion from a high quality evidence base. In the moderate and low quality arms, the qualitative conclusion can never be stronger than moderate or weak, respectively.

Figure 4 illustrates the branch followed when an evidence base has enough studies with calculable effect sizes to potentially reach a quantitative effect estimate, but the heterogeneity test indicates significant differences among the studies. If this heterogeneity can be explained using meta-regression, one can still reach a quantitative conclusion. The quantitative conclusion is the conclusion reached about the regression coefficients, including the intercept. For example, if gender is the variable that explains heterogeneity, one might have a conclusion such as "treatment X improved symptoms twice as effectively in women as in men". If meta-regression is not possible or does not explain heterogeneity, no quantitative conclusion is possible. However, one can still perform a random-effects meta-analysis which, if informative, may allow a qualitative conclusion.

Figure 5 illustrates what occurs when the evidence base is too small or otherwise insufficient to allow a quantitative conclusion. Some studies may not report effects sizes and standard errors (nor sufficient information for the analyst to calculate both measures). The analyst must acknowledge and adjust for the existence of such studies. This adjustment may require the estimation or imputation of effect sizes in certain studies [24]. The full evidence base is then assessed in a random-effects meta-analysis to determine if a qualitative conclusion can be reached.

If there are only two studies and both have calculable effect sizes, one performs a random-effects meta-analysis which, if informative, allows a qualitative conclusion. Of note, meta-analysis of a two-study evidence base is not required in the moderate quality arm, where a conclusion would require both studies to have a statistically significant effect. In the low quality arm, a minimum of three studies is required to reach any conclusion. A qualitative conclusion is also possible for two studies with imprecise effect sizes (that cannot be combined) if both studies are informative and show qualitatively consistent results. If two studies are qualitatively inconsistent or not informative when combined, the findings are inconclusive. If there is only one study, a large effect size is required to allow a weak qualitative conclusion (note that one cannot reach a conclusion if the single study is of moderate or low quality).

### Examples

In this section, we provide two example applications of our evidence rating system. In addition to illustrating various aspects of the system, we show how the system can be used in conjunction with simple declarative conclusions that are tied to the stability and strength ratings. The first example involves drug-eluting stents (DESs) for the treatment of coronary artery disease, and the second example involves positron emission tomography (PET) in the staging of lymphoma.

#### Example #1: Comparison between Drug-Eluting Stents and Bare-Metal Stents for the Treatment of Coronary Artery Disease

In a 2006 report, we examined the evidence comparing the safety and efficacy of drug-eluting stents (DESs) and bare-metal stents for the treatment of angina [13].

##### Evidence base

We included 14 randomized trials that compared drug-eluting stents to bare metal stents and reported the percentage of patients in each group who underwent target lesion revascularization (TLR) after stent implantation. The trials enrolled a total of 7,006 patients. We addressed the quantitative issue of the size of the difference in overall TLR rates and also the qualitative issue of whether there was any difference in overall TLR rates between the two types of stents.

##### Study quality assessment

To assess the quality of the studies, we applied a quality rating scale, and determined (using *a priori *definitions of high, medium, and low quality) that the evidence base was of high quality.

##### Sufficient data for quantitative estimate

In order to attempt a quantitative estimate of the effect, we required *a priori *that there must be at least three studies, and at least half of the included studies reported sufficient information for us to calculate effect sizes and confidence intervals. All 14 studies reported such information, so we attempted to make a quantitative estimate.

##### Heterogeneity testing

*A priori*, we defined quantitative consistency based on thresholds for Q and I^2^. These thresholds were a p value for the Q statistic less than 0.10 (which would mean quantitative inconsistency) and I^2 ^< 50% (which would also mean quantitative inconsistency). We used a p value of 0.10 because of the known low power of Q. We used a threshold of 50% for I^2 ^because this value represents moderate heterogeneity [19,20] For this evidence base, we performed a meta-analysis using Cohen's h and found substantial heterogeneity (I^2 ^= 78%, Q = 59, p value for Q was less than 0.000001).

##### Meta-regressions to explain heterogeneity

Given the heterogeneity of effect sizes, we performed meta-regressions in an attempt to explain why study results differed. Not all potential covariates could be examined because not all studies reported some covariates. The three covariates we could examine were drug type (paclitaxel or sirolimus), mean target vessel diameter, and mean target lesion length. None of these three factors were sufficient to explain the observed heterogeneity. Therefore, we did not draw a quantitative conclusion for the outcome of TLR rates. However, we proceeded to a qualitative analysis using a random-effects model to determine whether the evidence permitted a qualitative conclusion.

##### Stability rating

We rated the stability of the evidence as Unstable, due to the unexplained heterogeneity among effect sizes.

##### Informativeness

We performed a random-effects meta-analysis using Cohen's h. The summary statistic was statistically significant (lower TLR rates among patients who received DESs) and clinically important (because TLR is an important patient-oriented outcome, we defined clinical importance *a priori *as any statistically significant effect). This meant that the evidence was informative.

##### Qualitative robustness testing

*A priori*, we defined a quantitatively robust evidence base as one in which the confidence intervals of the last three cumulative, random-effects meta-analyses remained fully on the same side of zero after; 1) removal of the study with the smallest weight (i.e., the lowest precision), and, 2) after the additional removal of the study with the second smallest weight in the meta-analysis. Because the evidence base met both of these criteria, we deemed the evidence base to be qualitatively robust.

##### Strength rating

The strength rating of the evidence for a qualitative difference in TLR rates was Strong, because the meta-analysis was of high quality studies, and was informative and qualitatively robust.

##### Wording of conclusions

The conclusions for this outcome were phrased in the following manner:

The use of DESs (Cypher and TAXUS stents) leads to lower overall TLR rates than use of bare-metal stents in patients with angina at 6 to 12 months following stent implantation. (Strength of evidence: Strong)

• Due to unexplainable differences among the findings of different trials, one cannot accurately determine how much lower these rates are at 6 to 12 months following implantation of a DES.

#### Example #2: Positron Emission Tomography for the Staging of Lymphoma

In a 2006 report prepared by ECRI's Health Technology Assessment Information (HTAIS) under contract to TRICARE Management Activity (see Note 7), we assessed the use of positron emission tomography (PET) in the staging of lymphoma. The reference standard for determining whether lymphoma has reached Stage IV is a bone marrow biopsy typically taken from the iliac crest. PET may potentially help patients avoid the invasiveness of bone marrow biopsy, depending on how accurately it detects bone marrow infiltration.

##### Evidence base

We included five diagnostic cohort studies that performed PET as well as bone marrow biopsy and also reported sufficient information for the calculation of sensitivity and specificity. The studies reported data on a total of 243 patients. Because our research question was to determine a quantitative estimate of diagnostic accuracy, we did not attempt to draw a qualitative conclusion. Therefore, the text below refers only to the stability rating, not to a strength rating.

##### Study quality assessment

To assess the quality of the studies, we applied a quality rating scale, and determined (using *a priori *definitions of high, moderate and low quality) that the evidence base was of moderate quality. To rate the strength and stability of this evidence, we used the moderate quality branch of the system, which is not included in this paper, but is structurally very similar to the high quality arm. The key difference is an across-the-board decrease in both stability and strength ratings (e.g., "Strong" in the high quality branch corresponds to "Moderate" in the moderate quality branch).

##### Sufficient data for quantitative estimate

*A priori*, we decided that an evidence base could be considered sufficient to permit a quantitative estimate if there were at least three studies and also if at least 75% of the included studies had reported effect sizes or had provided sufficient information for the calculation of effect sizes. In this case, there were five studies, and both sensitivity and specificity were calculable for all five studies. Therefore, we proceeded with the quantitative analysis.

##### Heterogeneity testing

*A priori*, we defined quantitatively consistent results as an I^2 ^of less than 50% (see above). We computed the diagnostic odds ratio for each of the five studies, and the heterogeneity test revealed no heterogeneity (I^2 ^= 0%). Therefore, based on the definition of quantitative consistency, we deemed these findings to be quantitatively consistent. There was a threshold effect in the data, however, as evidenced by a plot of the data in ROC space and a strong negative correlation between sensitivity and specificity. We used the method of Littenberg and Moses [25] to construct a symmetric summary ROC curve, and computed a summary diagnostic odds ratio of 12.7 (95% confidence interval 5.5 to 29.7). At the mean threshold in the included studies, this estimate corresponded to a sensitivity of 64% and a specificity of 88%.

##### Informativeness

When evaluating this diagnostic, we attempted to reach only quantitative conclusions, not qualitative conclusions. Therefore, we did not consider informativeness, and we automatically proceeded to quantitative robustness testing for the summary diagnostic odds ratio (DOR).

##### Quantitative robustness testing

*A priori*, we defined a quantitatively robust evidence base as one that met both of the following two conditions: 1) the confidence interval around the summary DOR did not contain a DOR 50% higher or lower than the summary point estimate, and 2) a cumulative meta-analysis in which studies were entered by precision (highest precision study first) found that all of the last three analyses produced summary effects that were within 5% of the overall summary diagnostic odds ratio. In this case, the evidence base met neither of these conditions; therefore, we deemed the estimate to be not robust.

##### Stability rating

Using the system, we assigned a stability rating of Low to the estimated diagnostic odds ratio of 12.7 (95% CI 5.5 to 29.7). This rating was based on the fact that the studies were of moderate quality, and the estimate was not quantitatively robust.

##### Wording of conclusions

The conclusion was phrased in the following manner:

For the detection of bone marrow infiltration, at mean threshold PET has a sensitivity of 64% (95% CI: 43% to 80%), and a specificity of 88% (95% CI: 76% to 95%). Stability of estimate: Low.

## Discussion

The rating system described in this paper has several unique attributes. First, it distinguishes quantitative stability ratings from qualitative strength ratings. Second, it makes extensive use of *a priori *judgments to avoid bias. Third, it makes explicit the consequences of the results of meta-analysis and sensitivity analyses for both stability ratings and strength ratings. We have linked these attributes, and many others, into a logically consistent system intended to improve the process of systematic review.

Our system recognizes the fundamental role of judgment in summarizing evidence. The system is best viewed as a logical way to organize one's own judgments, not a predefined list of required judgments. Because different analysts can have different judgments, however, there is a critical need for transparency. This allows the user to trace the path of judgments that lead to a stability or strength rating. In fact, to use our system without making judgments explicit would constitute a misuse of the system.

Explicitness in judgments has been advocated by the GRADE group [2], but explicitness is not sufficient. Wherever possible, judgments should be made *a priori *and should be empirically-based. *A priori *judgments, a natural extension of *a priori *inclusion criteria, may help reduce bias by avoiding the influence of observed data on operational definitions. Also, basing judgments on empirical analyses, rather than opinion alone, may reduce bias. For example, judgments about quantity can be made empirically-based via formal sensitivity analysis.

While our system allows different analysts to make different judgments, it does place certain boundaries on judgments. If there are only one or two studies, for example, the evidence base must be judged insufficient to permit a quantitative estimate of the effect size. Further, if these one or two studies are of low quality, then no conclusion is possible. If there is only one study, then it must have been a high quality study observing a large effect in order to permit any conclusion. These limitations for small evidence bases are grounded in the principle of independent replication of scientific findings.

The GRADE system also places certain boundaries on the kinds of judgments permitted. In GRADE, one must give high priority to randomization: RCTs are initially judged as two levels stronger than observational studies, and three levels stronger than other study designs. Also, GRADE defines a "strong" association as a relative risk between 2 and 5 (or between 0.2 and 0.5) and a "very strong" association as a relative risk greater than 5 (or less than 0.2) (see Note 8). Other judgments are left up to the analyst, although GRADE recommends that all judgments be made explicit.

We next note some potential limitations of the system described in this paper. First, the system is complex. Understanding how it works, and more importantly why it works that way, requires careful study. Second, the system is resource-intensive, mostly because of the numerous *a priori *judgments. The analyst is responsible for making reasonable judgments before analyzing the data. For example, the *a priori *definition of a "clinically significant effect" is a critical judgment that can greatly impact the conclusions of the review. One strategy we have employed is to have additional clinicians and methodologists examine the analyst's *a priori *judgments for their reasonableness, and then to resolve disagreements in conference. These consensus judgments are then employed throughout the step-by-step system to produce evidence ratings. Whatever judgments are agreed upon, however, should be made fully explicit in the review. Such transparency, as emphasized throughout this paper, can enhance the flexibility and usefulness of the review.

In summary, explicit judgments and formal combination rules, as advocated by the GRADE group, represent two important steps on the path to a fully reliable system for rating medical evidence. Throughout this paper, we have argued that many additional steps must be taken. Foremost among these are distinguishing between quantitative and qualitative conclusions, making *a priori *judgments to avoid bias, and directly linking analytic results to evidence ratings.

Considered as a whole, our system constitutes a flexible tool for incorporating the full complexity of the evidence. Despite this complexity, the system outputs straightforward conclusions and ratings for medical decision makers to employ as they encounter difficult evidence-based decisions.

## Summary

• Systematic reviewers inevitably make judgments about the quality, quantity, consistency, robustness, and magnitude of effects observed in the studies identified

• This paper introduces a formal system for combining these judgments in a logical, consistent framework

• Unique aspects of the system include the distinction between quantitative and qualitative conclusions, extensive *a priori *criteria for judgments, and the direct impact of meta-analysis and sensitivity analysis on evidence ratings

• Stability ratings refer to the likelihood that future evidence will indicate a different size of effect

• Strength ratings refer to the likelihood that future evidence will overturn conclusions about whether a device, drug or procedure is effective (or harmful)

## Competing interests

The author(s) declare that they have no competing financial interests. JRT, SJT, and JTR are each employed by the ECRI Health Technology Assessment Information Service, which produces systematic reviews. ECRI paid the article processing charge for this manuscript.

## Authors' contributions

All authors made substantial contributions to the ideas presented in this manuscript. JT contributed extensively to the development of the rating system, devised the manuscript outline, and wrote the manuscript. CT supervised the project and contributed extensively to developing and testing the system. ST originated the idea for the rating system and contributed extensively to its development. JR contributed through editing and testing the system, and wrote the section entitled "How the System Works". All authors participated in the editing process, and all authors read and approved the final manuscript.

## Notes

1 – Throughout this paper, the word "technology" is used generically to refer to drugs, devices, or procedures.

2 – The specific outcome could be a surrogate outcome or a patient-oriented outcome.

3 – The presence of heterogeneity is a violation of the assumption of fixed-effects models: that the available data are sampled from a single distribution that describes a single summary estimate of treatment effect.

4 – The use of 0 assumes that the effect size metric is centered around 0 (e.g., the standardized difference between means). Other effect size metrics (e.g., relative risk, odds ratio) are centered around 1, and for these one would determine whether the random-effects confidence interval were fully above 1 or below 1.

5 – We refer to an entire family of procedures designed to assess the sensitivity of one's conclusions to certain aspects of the data or certain analytic assumptions. Many of these were listed in a paper by Olkin,[26] and some examples include publication bias testing, [27-29] cumulative meta-analysis, [21,30] and the removal of specific subsets of the data.

6 – Although not illustrated in the figure, there are possible effect sizes corresponding to A through D that would fall on the left side of the graph (below zero). Some of these possibilities would lead to a conclusion that the treatment was either harmful or inferior to a comparison treatment.

7 – TRICARE is the government agency under the Department of Defense responsible for administering the health benefits for the U.S. armed forces and their dependants.

8 – Note that the GRADE system's treatment of effect magnitude does not specifically mention the confidence interval around the effect. Instead the system addresses imprecise or sparse data in a separate manner. Our system considers an effect size and its confidence interval simultaneously.

## Pre-publication history

The pre-publication history for this paper can be accessed here:


